# Magnetic Properties of High-Entropy Alloy FeCoNiTi

**DOI:** 10.1021/acsomega.4c04556

**Published:** 2024-08-22

**Authors:** Jiro Kitagawa, Daiki Shintaku

**Affiliations:** Department of Electrical Engineering, Faculty of Engineering, Fukuoka Institute of Technology, Fukuoka 811-0295, Japan

## Abstract

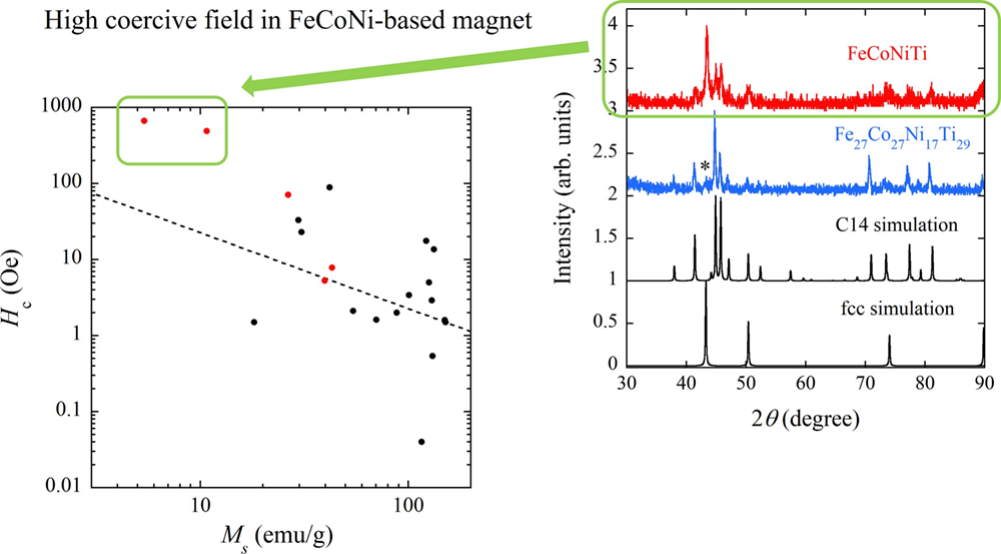

We report the magnetic properties of the as-cast high-entropy alloy (HEA) FeCoNiTi, characterized by a dual phase comprising the face-centered cubic (fcc) and hexagonal C14 Laves phases. The HEA manifests three distinct ferromagnetic orderings at 1084, 214, and 168 K. The emergence of the 214 K transition is attributed to the influence of the C14 phase. The high-temperature ordering at 1084 K is associated with the fcc phase, which exhibits an additional ferromagnetic ordering at 168 K. The coercive field of the fcc phase attains 667 Oe at 400 K. Electronic structure calculations conducted for both phases substantiate the presence of ferromagnetic ground states. Comparative analyses between experimental and theoretical values are undertaken in the context of saturation magnetization. A comprehensive discussion is presented, delving into the origin of the relatively high coercive field observed at high temperatures in the fcc phase.

## Introduction

A high-entropy alloy (HEA) is delineated as an alloy comprising multiple principal elements with an almost equiatomic composition.^1,2^ The thermodynamic evaluation of alloy entropy is executed through configurational entropy, denoted as Δ*S*_mix_ = −*R*∑ _*i* = 1_^*n*^*c*_*i*_ln*c*_*i*_, where *n* represents the number of elements, *c*_*i*_ denotes the atomic fraction, and *R* is the gas constant. The established threshold value of Δ*S*_mix_, defining HEA, is presently set at 1.0 *R*, typically achieved by incorporating four principal elements.^3^ HEAs exhibit significant promise for practical applications due to their exceptional attributes, encompassing high strength and ductility, corrosion resistance, energy storage capabilities, magnetic refrigeration, soft ferromagnetism, thermoelectricity, and superconductivity.^4−13^

The expansive compositional domain of HEAs affords us more freedom in designing single-phase alloys with novel properties.^14,15^ Furthermore, the vast number of elemental combinations gives rise to multiphase HEAs, contributing to their multifaceted functionalities. A prominent illustration is the dual-phase HEAs featuring face-centered cubic (fcc) and body-centered cubic (bcc) phases, which exhibit a superior balance of strength and ductility.^16^ While singe-phase bcc HEAs display limited ductility, fcc HEAs with single-phase showcase elevated ductility and diminished strength. This trade-off between strength and ductility can be surmounted by incorporating both bcc and fcc phases within HEAs. In a magnetic HEA characterized by multiple phases, each with a distinct magnetic ground state independently shows respective magnetic phenomenon. For instance, a dual-phase Al_*x*_FeCoNiCr (*x* = 1.0, 1.25, and 1.75) comprises a bcc (Fe–Cr rich) phase and a B2 (Al-(Ni, Co) rich) phase, elucidating weak ferromagnetic and strong ferromagnetic correlations, respectively.^17^ Another example is FeRhIrPdPt, which shows a mixture of two fcc phases with different chemical compositions.^18^ The major fcc phase is rich in Rh and Ir, while the minor one is enriched in Pd and Pt. Notably, two magnetic properties, a canonical spin-glass transition and a ferromagnetic correlation, manifest in FeRhIrPdPt. The spin-glass transition and the ferromagnetic correlation are attributed to the main and minor phases, respectively.^18^

A recent report highlights the soft ferromagnetism in single-phase fcc HEAs, specifically FeCoNiPd and FeCoNiPt.^19^ These HEAs can be construed as Pd- or Pt-added derivatives of the FeCoNi alloy. FeCoNi adopts the fcc structure, displaying a high saturation magnetization (*M*_s_) and a low coercive field (*H*_c_).^20^ Various elements can be incorporated into FeCoNi while maintaining a singe-phase fcc structure.^11,21^ To our knowledge, single-phase fcc HEAs derived from FeCoNi exhibit *H*_c_ values that do not surpass 100 Oe. Consequently, we aim to investigate an fcc HEA based on FeCoNi with an *H*_c_ surpassing 100 Oe, encompassing both single-phase and multiphase HEAs. Our attention is directed toward FeCoNiTi, an alloy lacking reported magnetic properties. An initial investigation into FeCoNiTi has unveiled enhanced mechanical properties resulting from thermal annealing.^22^ The as-cast sample exhibits a two-phased dendritic microstructure featuring dendrites of hexagonal structure and an fcc interdendritic matrix.^22^ Following annealing at 1000 °C for 24 h, the sample demonstrates exceptional mechanical characteristics, including a yield strength of 1.33 GPa and an ultimate compressive strength of 2.6 GPa. A recent study by Liu et al. provides comprehensive structural and compositional analyses of FeCoNiTi.^23^ According to their findings, the as-cast FeCoNiTi manifests a dual-phase microstructure comprising fcc and hexagonal C14 Laves phases, with average elemental compositions of Fe_17_Co_27_Ni_32_Ti_23_ for the fcc phase and Fe_32_Co_29_Ni_11_Ti_28_ for the C14 phase, respectively.^23^

This paper demonstrates the magnetic properties of as-cast FeCoNiTi. The discernment of magnetic characteristics within the fcc and hexagonal C14 phases was achieved by synthesizing an alloy dominated by the C14 phase. FeCoNiTi exhibits three distinct ferromagnetic transitions, wherein one is attributed to the influence of the C14 phase, while the fcc phase induces the remaining two. *H*_c_ attains a magnitude of 667 Oe at 400 K within the fcc phase. Comparative analysis of the *M*_s_ values of the fcc and C14 phases is conducted by computations using an electronic structure calculation program. We scrutinize the origin of the relatively high *H*_c_ through a survey of the magnetic properties of typical single-phase fcc HEAs based on FeCoNi.

## Materials and Methods

The as-cast polycrystalline sample (1.5 g) of FeCoNiTi was prepared through a homemade arc furnace. The elemental components, Fe chips (Kojundo Chemical Laboratory, 99.9%), Co chips (Kojundo Chemical Laboratory, 99.9%), Ni wire (Soekawa Rikagaku, 99.9%), and Ti wire (Nilaco, 99.9%), were employed in a fixed atomic ratio of Fe:Co:Ni:Ti = 1:1:1:1. These constituent elements were remelted several times on a water-cooled Cu hearth and flipped each time to ensure homogeneity under Ar atmosphere. The finalization of the sample preparation involved rapid quenching on the water-cooled Cu hearth.

Room temperature X-ray diffraction (XRD) patterns were captured utilizing an X-ray diffractometer (XRD-7000L, Shimadzu) with Cu–Kα radiation in the Bragg–Brentano geometry. Scanning electron microscopy (SEM) images were acquired using a field-emission scanning electron microscope (FE-SEM, JSM-7100F, JEOL), focusing on the polished surfaces. The preparation of the FeCoNiTi sample surface involved successive polishing using silicon carbide paper (#240, #400, #600, and #1000 mesh), alumina paste (5, 1, and 0.1 μm), and colloidal silica (0.04 μm). Chemical composition evaluation was performed using an energy-dispersive X-ray (EDX) spectrometer colocated with the FE-SEM.

The temperature-dependent DC magnetization χ_dc_ (*T*) ranging from 50 to 400 K was recorded utilizing the VersaLab apparatus (Quantum Design). Isothermal magnetization (*M*) curves were obtained employing the same equipment. For χ_dc_ (*T*) measurements from 400 to 1150 K, a vibrating sample magnetometer (TM-VSM33483-HGC, Tamakawa) was employed.

Electronic structure calculations were also executed utilizing the coherent potential approximation (CPA) approach. To this end, the Akai-KKR program package,^24^ grounded in the Korringa–Kohn–Rostoker (KKR) method with CPA, was deployed. Based on Green’s function and multiple scattering principles, this program obviates the need for a supercell structure in handling chemically disordered materials. We used the Perdew–Burke–Ernzerhof (PBE) exchange-correlation potential and treated the spin-polarization and the spin–orbit interaction.

As mentioned in the next section, the as-cast FeCoNiTi is composed of the fcc and hexagonal C14 phases with the volume ratio of *V*_fcc_:*V*_C14_ = 57:43. Chemical composition analysis indicates that the fcc phase is Fe_21.3_Co_24.8_Ni_32.2_Ti_21.6_, while the C14 phase is Fe_27.3_Co_26.6_Ni_17.4_Ti_28.7_. Both phases undergo ferromagnetic (FM) ordering. To determine which phase is responsible for each ordering, we prepared as-cast Fe_27_Co_27_Ni_17_Ti_29_, corresponding to Fe_27.3_Co_26.6_Ni_17.4_Ti_28.7_ of the C14 phase in FeCoNiTi, using an analogous procedure. As anticipated, Fe_27_Co_27_Ni_17_Ti_29_ is predominantly composed of the C14 phase. Comparing the magnetic properties of FeCoNiTi and Fe_27_Co_27_Ni_17_Ti_29_ elucidates which phase is responsible for the respective ordering temperatures.

## Results and Discussion

**Figure 1:**
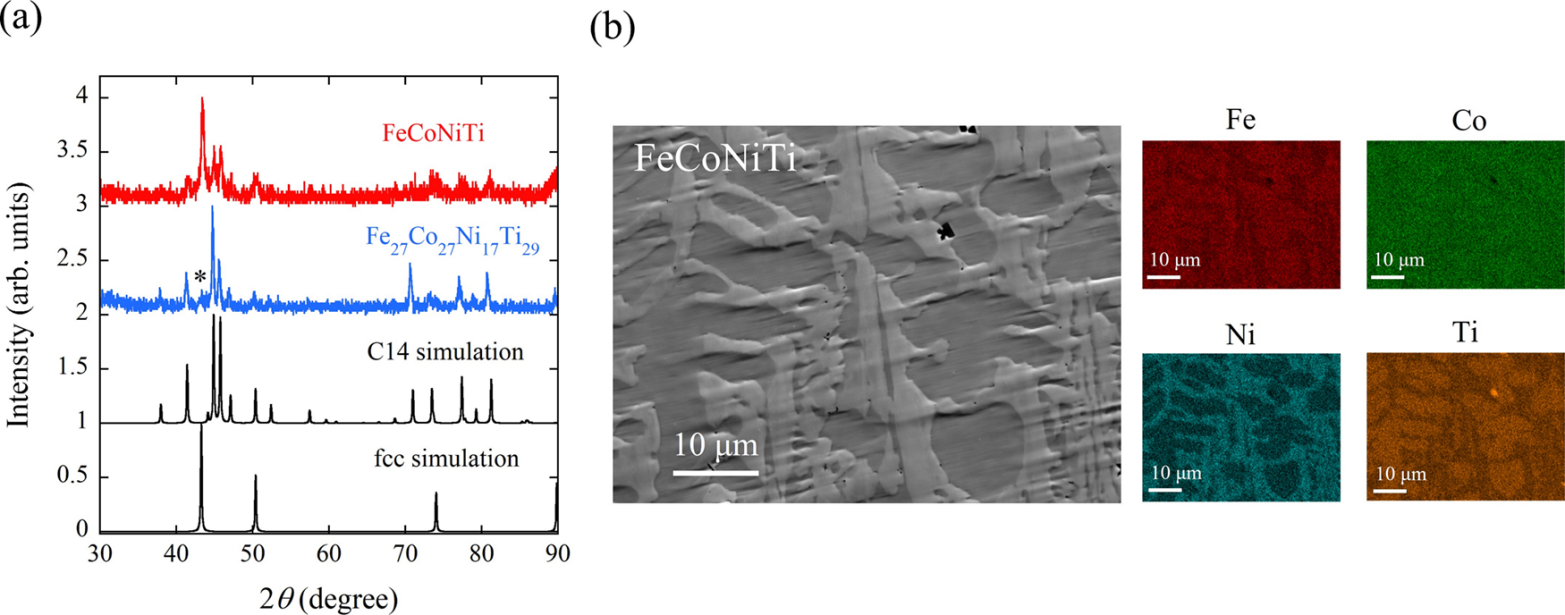
(a) XRD patterns of FeCoNiTi and Fe_27_Co_27_Ni_17_Ti_29_ in as-cast states. The simulated patterns of fcc (*a* = 3.618 Å) and C14 (*a* = 4.731 Å and *c* = 7.705 Å) phases are also shown. The origin of each pattern is shifted by a value for clarity. (b) SEM image of FeCoNiTi. The elemental mappings are also displayed.

Figure 1a depicts the XRD pattern of FeCoNiTi. In accordance with the findings elucidated by Liu et al.,^23^ the Bragg reflection positions of FeCoNiTi can be indexed by the fcc or hexagonal C14 Laves structure. The lattice parameters of the fcc and C14 phases were determined through the least-square method, yielding *a* = 3.618 Å for the fcc phase and (*a* and *c*) = (4.731 and 7.705 Å) for the C14 phase, respectively. Figure 1b presents the SEM image of FeCoNiTi, unveiling two distinct phases; the bright and dark regions correspond to the fcc and C14 phases, respectively. The volume fraction ratio is discerned as *V*_fcc_:*V*_C14_ = 57:43 based on the SEM image. This ratio is calculated through the equations *V*_fcc_ = (*A*_fcc_)^1.5^ and *V*_C14_ = (*A*_C14_)^1.5^, where *A*_fcc_ and *A*_C14_ are the areas of the fcc and C14 phases estimated from the SEM image. Elemental mappings in Figure 1b indicate that the bright and dark phases are enriched in Ni and Ti, respectively. Atomic compositions, as evaluated by EDX in the bright and dark regions, are Fe_21.3(6)_Co_24.8(3)_Ni_32.2(3)_Ti_21.6(2)_ and Fe_27.3(2)_Co_26.6(4)_Ni_17.4(2)_Ti_28.7(1)_, respectively. These findings align with the literature by Liu et al.,^23^ affirming that the fcc phase corresponds to Fe_17_Co_27_Ni_32_Ti_23_, while the C14 phase corresponds to Fe_32_Co_29_Ni_11_Ti_28_. Therefore, the bright and dark regions in the SEM image are unequivocally attributed to the fcc and C14 phases, respectively. XRD patterns for the fcc and C14 phases were simulated utilizing the obtained chemical compositions and lattice parameters. In the fcc phase, the four atoms are randomly distributed in the Wyckoff position denoted as 4*a* within the cubic structure with the space group of  (No. 225). The C14 Laves phase adopts a hexagonal structure (space group: *P*6_3_/*mmc*, No. 194), comprising three inequivalent sites with the 2*a*, 4*f*, and 6*h* designations. Full occupation of Ti atoms at the 4*f* site and the random occupation of Fe, Co, and Ni atoms at the 2*a* and 6*h* sites were assumed. The simulated patterns for the fcc and C14 Laves phases are juxtaposed with the experimental XRD pattern of FeCoNiTi in Figure 1a. The experimental XRD pattern undeniably incorporates the simulation patterns of both phases. To disentangle the magnetic properties of the fcc and C14 phases, we synthesized Fe_27_Co_27_Ni_17_Ti_29_, corresponding to the chemical composition ascertained for the C14 phase. Figure 1a also illustrates the XRD pattern of this alloy, primarily dominated by the XRD pattern of the C14 phase, albeit with the presence of the fcc phase as a minor component (indicated by * in Figure 1a). We comment here on the configurational entropy of the fcc phase (Fe_21.3_Co_24.8_Ni_32.2_Ti_21.6_). Δ*S*_mix_ calculated using the equation denoted in the Introduction is 1.37 *R*, which exceeds the recent threshold value of 1.0 *R*, defining the HEA state.^3^

**Figure 2:**
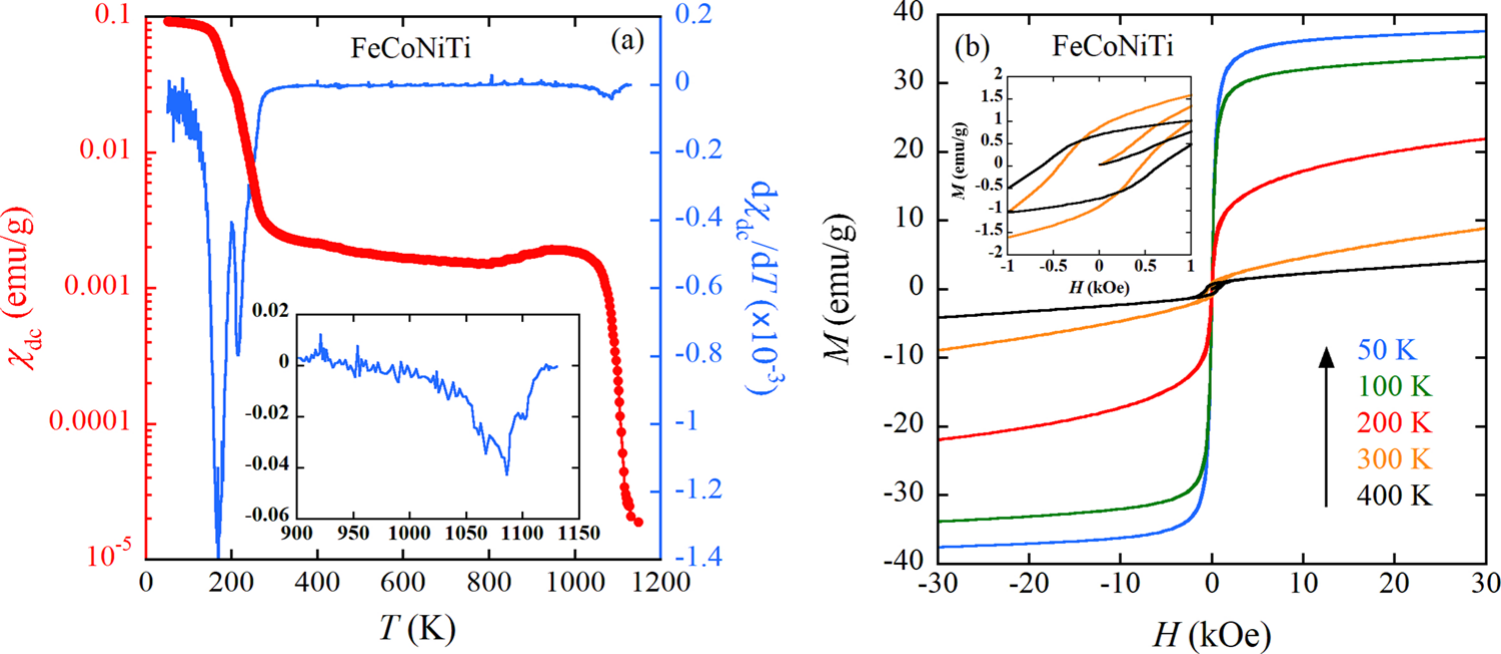
(a) Temperature dependences of χ_dc_ and temperature derivative of χ_dc_ of as-cast FeCoNiTi. The external field is 100 Oe. The inset is the expanded view of the temperature derivative of χ_dc_ at high temperatures. (b) Isothermal magnetization curves at 50, 100, 200, 300, and 400 K of as-cast FeCoNiTi. The inset is the expanded view at the low-field region for data acquired at 300 and 400 K.

Figure 2a illustrates χ_dc_(*T*) under an external field *H* of 100 Oe for FeCoNiTi, employing a logarithmic scale for χ_dc_. As the temperature decreases from 1150 K, χ_dc_ sharply rises around 1100 K, followed by a plateau extending to approximately 300 K. Subsequent cooling prompts a second notable increase in χ_dc_. These outcomes suggest the presence of at least two FM phases in FeCoNiTi, a phenomenon explicable by FM orderings in both the fcc and C14 phases. To ascertain the Curie temperature (*T*_c_), the temperature derivative of χ_dc_ is depicted in Figure 2a. The local minima of *d*χ_dc_/*dT* define the *T*_c_ values, a methodology widely applied in analyzing transition metal-based alloys and compounds.^25−27^ The thus-obtained *T*_c_ values are 1084, 214, and 168 K. The subsequent discussion delves into assigning two phases to their respective ordering temperatures. The comparison of χ_dc_(*T*) between FeCoNiTi and C14 phase dominant Fe_27_Co_27_Ni_17_Ti_29_ suggests that the FM orderings at *T*_c_ = 1084 and 168 K occur in the fcc phase and that at *T*_c_ = 214 K is induced by the C14 phase (see also the next paragraph). Magnetization curves at 50, 100, 200, 300, and 400 K are presented in Figure 2b. All curves consistently manifest a FM state within the investigated temperature range. Preceding the onset of low-temperature FM ordering at 214 K, the *M*–*H* curves at 300 and 400 K exhibit distinct hysteresis loops, albeit with less elevated high-field *M* values (refer to the inset of Figure 2b). *H*_c_ experiences a rapid deterioration in the low-temperature FM state; inversely, *M* escalates as the temperature descends below 300 K.

**Figure 3:**
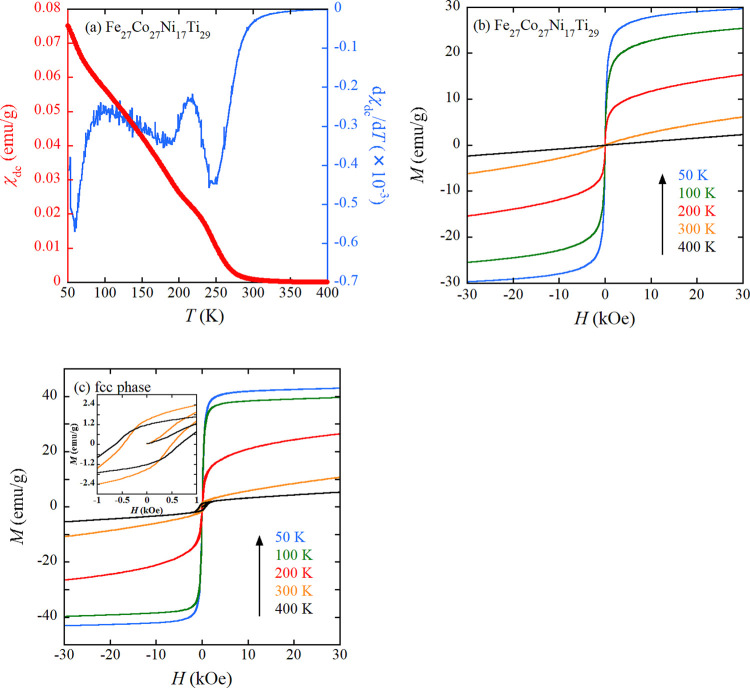
(a) Temperature dependences of χ_dc_ and temperature derivative of χ_dc_ of as-cast Fe_27_Co_27_Ni_17_Ti_29_ dominated by C14 phase. The external field is 100 Oe. (b) Isothermal magnetization curves at 50, 100, 200, 300, and 400 K of as-cast Fe_27_Co_27_Ni_17_Ti_29_. (c) Isothermal magnetization curves at 50, 100, 200, 300, and 400 K of fcc phase extracted using eq 1. The inset is the expanded view at the low-field region for data acquired at 300 and 400 K.

Figure 3a shows the χ_dc_(*T*) of Fe_27_Co_27_Ni_17_Ti_29_, dominated by the C14 phase, measured under *H* of 100 Oe. The increase of χ_dc_ below approximately 275 K signifies a FM ground state. The temperature derivative of χ_dc_ is concurrently illustrated in Figure 3a, revealing three distinct anomalies at 246, 183, and 58 K. Figure 3b showcases the magnetization curves of Fe_27_Co_27_Ni_17_Ti_29_ at the same temperatures as those explored in the case of FeCoNiTi. At 50 K, well below the highest *T*_c_ of 246 K, Fe_27_Co_27_Ni_17_Ti_29_ exhibits a characteristic *M*–*H* curve indicative of ferromagnetism. Conversely, the *M–H* curve at 300 K suggests a paramagnetic behavior, signifying that the high-temperature ferromagnetism observed above 300 K in FeCoNiTi originates from the fcc phase. In the temperature range from 100 to 300 K, *d*χ_dc_/*dT* for Fe_27_Co_27_Ni_17_Ti_29_ reveals two anomalies at 183 and 246 K. The bifurcated nature of these anomalies, akin to that observed in *d*χ_dc_/*dT* of FeCoNiTi, is noteworthy. However, the low-temperature anomaly in FeCoNiTi is more pronounced compared to Fe_27_Co_27_Ni_17_Ti_29_. In the latter, despite the predominant presence of the C14 phase, the fcc phase persists. Consequently, the FM ordering at 168 K in FeCoNiTi is intrinsically linked to the fcc phase, while the C14 phase in FeCoNiTi governs the ferromagnetic ordering at 214 K. The magnetic transition at 58 K in Fe_27_Co_27_Ni_17_Ti_29_ is likely attributable to a parasitic phase. In summation, two of the three FM orderings in FeCoNiTi (*T*_c_ = 1084 and 168 K) are induced by the fcc phase, with the ferromagnetic ordering at 214 K being attributed to the C14 phase.

To evaluate the magnetic contribution solely from the fcc phase, *M*–*H* curves originating from the fcc phase are extracted through the following procedure. For FeCoNiTi, comprising fcc (57% vol.) and C14 (43% vol.) phases, the weight fraction ratio is determined as fcc:C14 = 59:41, leveraging density values of 7.8241 g/cm^3^ for fcc and 7.2632 g/cm^3^ for C14. Subsequently, assuming that the *H* dependence of *M* (*M*(*H*)) of the C14 phase mirrors that of Fe_27_Co_27_Ni_17_Ti_29_, *M*(*H*) of the fcc phase at a fixed temperature can be computed using the equation:
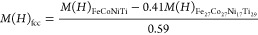
1where *M*(*H*)_FeCoNiTi_ and *M*(*H*)_Fe_27_Co_27_Ni_17_Ti_29__ represent the magnetization of FeCoNiTi and Fe_27_Co_27_Ni_17_Ti_29_, as illustrated in Figures 2b and 3b, respectively. The deduced *M*(*H*) of the fcc phase is exhibited in Figure 3c. The high-field magnetization experiences a rapid augmentation as the temperature descends from 300 to 100 K, suggesting the consistency with additional FM ordering in the fcc phase, although a more detailed investigation is required. At 300 and 400 K, *H*_c_ reaches 489 and 667 Oe, respectively (refer to the inset of Figure 3c), categorizing the fcc Fe_21_Co_25_Ni_32_Ti_22_ above 300 K as high *H*_c_ materials within the realm of fcc HEAs based on FeCoNi.

**Figure 4:**
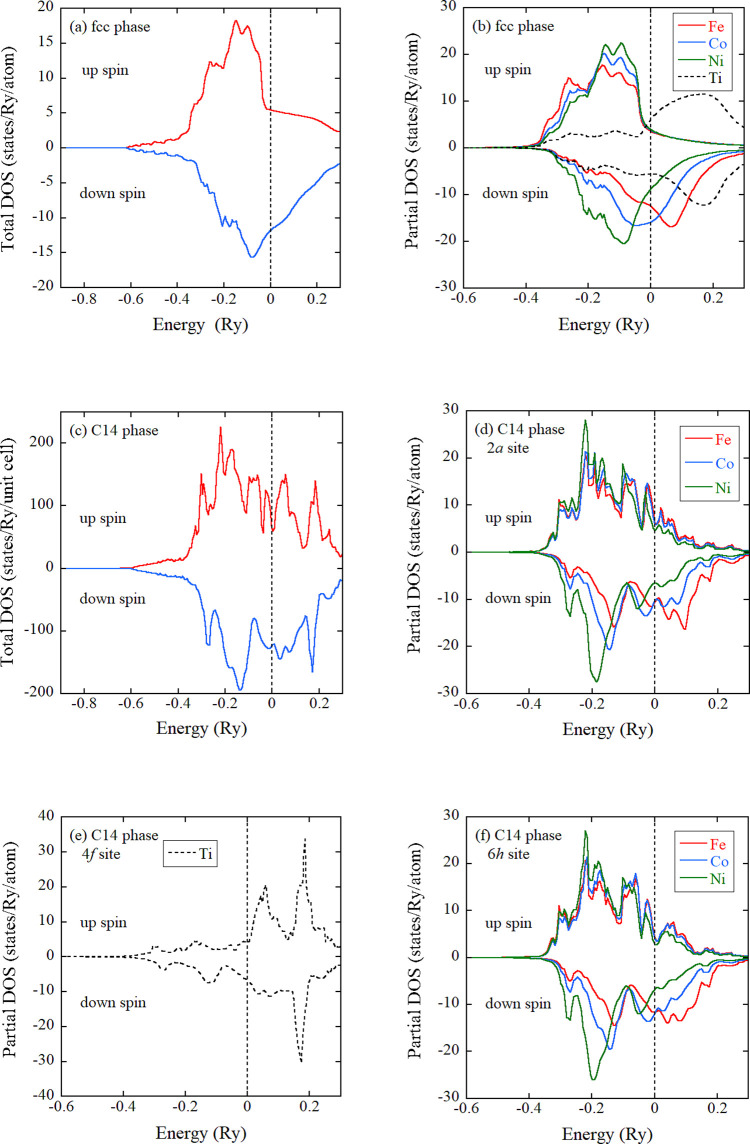
Electronic total density of states (DOS) of (a) fcc and (c) C14 phases. (b) Partial DOS of fcc phase. (d) Partial DOS at 2*a* site of C14 phase. (e) Partial DOS at 4*f* site of C14 phase. (f) Partial DOS at 6*h* site of C14 phase. Each partial DOS is drawn for only *d*-electrons due to the dominant contribution around the Fermi level. The Fermi energy is set to 0 Ry.

Electronic structure calculations were executed to elucidate the ferromagnetic characteristics inherent in each phase, and the outcomes are delineated in Figure 4a–f. Figure 4a,b presents the total density of states (DOS) and partial DOSs specifically for the fcc phase. The chemical composition of the fcc phase is denoted by Fe_21_Co_25_Ni_32_Ti_22_, as determined by EDX analysis, and the experimental lattice parameter is employed. In Figure 4b, the *d*-electron DOS is illustrated for each element, as it predominates over *s*- or *p*-electron DOS at the Fermi energy (*E*_F_). The disparate energy dependence of total DOS between spin-up and spin-down at *E*_F_ substantiates the FM ground state in the fcc phase. The partial DOSs elucidate the detailed magnetic features of constituent elements in Figure 4b. Progressing from Fe, Co to Ni, the occupancy below *E*_F_ for the down spin increases, while that for the up spin remains nearly constant in a filled state. This signifies that the magnetic moment is the largest for the Fe atom and the smallest for the Ni atom, as listed in Table 1. Notably, these atoms exhibit FM coupling to each other. Regarding the Ti atom, the *d*-electron DOS below *E*_F_ is relatively modest compared to the other elements. The calculated magnetic moment of Ti is not exceedingly large and is antiferromagnetically coupled to Fe, Co, and Ni atoms. Figure 4c presents the total DOS for the C14 phase, characterized by the chemical composition as Fe_27_Co_27_Ni_17_Ti_29_. The discrepancy in total DOS between spin-up and spin-down configurations posits the manifestation of a FM ground state, concordant with our experimental findings. The elemental dependence of *d*-electron DOS at the 2*a* and 6*h* sites manifests a similar pattern; notably, the down spin peak systematically shifts to lower energy levels in the progression from Fe, through Co, to Ni (see Figure 4d,f). The *d*-electron DOS beneath *E*_F_ of Ti at the 4*f* site demonstrates relative diminution when compared with other constituent elements, as elucidated in Figure 4e. Table 1 compares spin moments for the constituent elements in the fcc phase with their counterparts in the C14 phase. In both phases, the magnetic moments of Fe, Co, and Ni exhibit ferromagnetic alignment, while the orientation of the Ti moment is antiparallel to that of Fe, Co, or Ni. Notably, within each elemental category, the absolute magnitude of the magnetic moment in the fcc phase exceeds that obtained in the C14 phase.

**Table 1:** Spin Moment and Orbital Moment of Each Element in fcc and C14 Phases Obtained by Electronic Structure Calculation^a^

fcc			C14		
atom	spin moment (μ_B_)	orbital moment (μ_B_)	atom	spin moment (μ_B_)	orbital moment (μ_B_)
Fe	2.254	0.0619	Fe (2*a*)	1.364	0.0474
Co	1.285	0.0727	Co (2*a*)	0.688	0.0453
Ni	0.341	0.0236	Ni (2*a*)	0.209	0.0160
Ti	–0.426	0.0105	Ti (4*f*)	–0.381	0.0101
			Fe (6*h*)	1.428	0.0481
			Co (6*h*)	0.663	0.0407
			Ni (6*h*)	0.151	0.0108

a The atomic compositions of fcc and C14 phases are Fe_21_Co_25_Ni_32_Ti_22_ and Fe_27_Co_27_Ni_17_Ti_29_, respectively. The total magnetic moments are 0.7843 μ_B_/f.u. and 4.8407 μ_B_/f.u. for the fcc and C14 phases, respectively.

Subsequently, we assessed *M*_s_ and compared it with the experimental counterpart in each phase. By employing the total moment value of 4.8407 μ_B_/f.u. for the C14 phase derived from the band calculation, *M*_s_ in the unit of emu/g is determined as
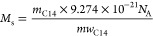
2where *m*_C14_, *mw*_C14_, and *N*_A_ denote the theoretical total moment, the molecular weight of the C14 phase corresponding to the total moment, and the Avogadro number, respectively. In the C14 phase, the total moment value pertains to the formula unit encompassing 12 atoms (2 atoms in the 2*a* site, 4 atoms in the 4*f* site, and 6 atoms in the 6*h* site). Consequently, *mw*_C14_ is computed as 658.193 g. The resultant *M*_s_ value is 41.1 emu/g, in roughly agreement with the experimental value of 29.7 emu/g at 50 K and 30 kOe (see also Figure 3b). In the fcc phase, the theoretical *M*_s_ is ascertained to be 78.6 emu/g, employing the identical equation (eq 2), with the molecular weight of the fcc phase being 55.77 g. As depicted in Figure 3c, the high-field *M* at 300 K is 10.8 emu/g, markedly smaller than the anticipated theoretical *M*_s_. Upon cooling to 50 K, the high-field *M* escalates to 43 emu/g, leading to a notable diminution in the disparity between experimental and theoretical values. Given that theoretical values are typically acquired at 0 K, the electronic structure calculation lends credence to the existence of low-temperature FM ordering in addition to the high-temperature counterpart at 1084 K. In both the fcc and C14 phases, theoretical *M*_s_ values surpass their experimental counterparts. The band calculation treats the FM state with spins of Fe, Co, and Ni aligning in parallel, while the Ti spin aligns antiparallel. Therefore, the diminished experimental *M*_s_ in each phase signifies the presence of a spin-canted component within the actual magnetic structure.

We discuss the origin of the relatively high *H*_c_ observed in the fcc phase above 300 K. Initially, we focus on the orbital moment, a determinant of the anisotropy in magnetic interactions and a factor influencing *H*_c_. Notably, in Fe_1/4_TaS_2_, the Fe atom exhibits a substantial unquenched orbital magnetic moment of 1 μ_*B*_/Fe, resulting in a giant *H*_c_ surpassing 20 kOe at 2 K.^28^ To gauge the impact of the orbital moment on *H*_c_ in the fcc phase, we conducted electronic structure calculations for the isostructural FeCoNi. FeCoNi is characterized by soft ferromagnetism, showing an *H*_c_ of 1.5 Oe at room temperature.^20^ Should the fcc Fe_21_Co_25_Ni_32_Ti_22_ exhibit orbital moments greater than those of FeCoNi, the heightened *H*_c_ in fcc Fe_21_Co_25_Ni_32_Ti_22_ can be attributed to the orbital moments of its magnetic constituents. For FeCoNi, the spin and orbital moments are determined as 2.604 μ_B_ and 0.0640 μ_B_ for Fe, 1.698 μ_B_ and 0.0876 μ_B_ for Co, and 0.7002 μ_B_ and 0.0484 μ_B_ for Ni. In each elemental component, both spin and orbital moments are greater for FeCoNi, signifying that the orbital moment in the fcc phase does not yield an augmented *H*_c_.

**Figure 5:**
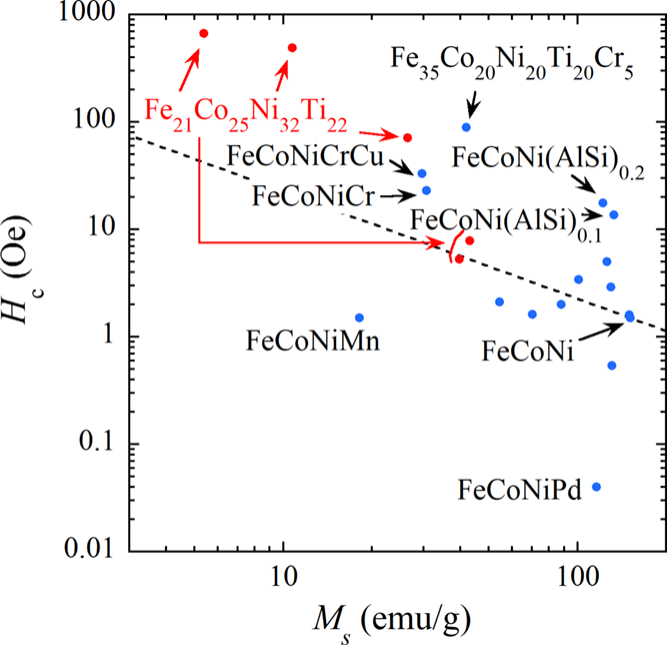
*H*_c_ vs *M*_s_ plot for typical fcc magnetic HEAs (blue) based on FeCoNi and experimental data of present work (red). The numerical data of all alloys are listed in Table 2. The dotted line is a guide representing *H*_c_ = 2*K*/μ_0_*M*_s_ with *K* = 93.5 J/m^3^.

Within the fcc Fe_21_Co_25_Ni_32_Ti_22_, the decrease in temperature leads to a reduction in *H*_c_ and concurrent augmentation of high-field magnetization, as depicted in Figure 3c. This observation implies a negative correlation between *H*_c_ and *M*_s_. Following the claim of a simple magnetic anisotropy model, *H*_c_ is described as 2*K*/μ_0_*M*_s_, where *K* represents the magnetocrystalline anisotropy constant, and μ_0_ is the magnetic permeability of vacuum.^29^ Consequently, we expect that the magnetocrystalline anisotropy predominantly governs *H*_c_ within the fcc phase. To check the inverse relationship between *H*_c_ and *M*_s_, we constructed a *H*_c_ vs *M*_s_ plot with double logarithmic scales for representative fcc magnetic HEAs, as illustrated in Figure 5. This plot incorporates experimental data (red) extracted from Figure 3c, with detailed numerical data provided in Table 2. The fcc HEAs exhibit a potentially universal behavior, wherein *H*_c_ intensifies with decreasing *M*_s_. We have evaluated *K* using the *H*_c_ and *M*_s_ data set of FeCoNi listed in Table 2. The *K* value is extracted as 93.5 J/m^3^. The dotted line in Figure 5 serves as a guide, depicting *H*_c_ = 2*K*/μ_0_*M*_s_ with *K* = 93.5 J/m^3^. Although the slope of the data set for fcc HEAs exhibits a slight deviation from that of the guideline, it is apparent that magnetocrystalline anisotropy partially accounts for the observed experimental trend. Our discussion illuminates a strategic avenue for augmenting *H*_c_ in fcc magnetic HEAs based on FeCoNi: the deliberate reduction of *M*_s_. We further discuss the effect of elements added to FeCoNi on the magnitude of *H*_c_ and *M*_s_. As noted in Figure 5, Ti addition leads to relatively high *H*_c_, as represented by Fe_21_Co_25_Ni_32_Ti_22_ and Fe_35_Co_20_Ni_20_Ti_20_Cr_5_. The Cr-added FeCoNiCrCu and FeCoNiCr occupy a moderately higher *H*_c_ region. The Ti (or Cr) addition yields antiferromagnetic coupling between FeCoNi and Ti (or Cr), which reduces *M*_s_. In FeCoNi(AlSi)_0.1_ and FeCoNi(AlSi)_0.2_, in which Al and Si atoms are simultaneously added, *H*_c_ is not significantly reduced, while *M*_s_ is comparable to that of FeCoNi. For the lower *H*_c_ region, FeCoNiMn and FeCoNiPd are noteworthy. In FeCoNiMn, antiferromagnetic coupling between FeCoNi and Mn is induced, but *H*_c_ is not substantially enhanced. Based on the discussion concerning the effect of atomic species added to FeCoNi, the addition of Ti might be crucial for enhancing *H*_c_.

**Table 2:** *H*_c_ and *M*_s_ of Typical fcc Magnetic HEAs Based on FeCoNi and fcc Phase of Present Study

alloy	*H*_c_ (Oe)	*M*_s_ (emu/g)	reference
FeCoNi	1.5	151.3	(20)
FeCoNiAl_0.2_	0.54	131	(30)
FeCoNiAl_0.25_	2.9	130	(20)
FeCoNiSi_0.25_	5.0	126	(20)
FeCoNiAl_0.25_Mn_0.25_	3.4	101	(31)
FeCoNi(AlMn)_0.1_	1.6	150	(32)
FeCoNi(AlSi)_0.1_	13.7	133	(33)
FeCoNi(AlSi)_0.2_	17.6	122	(33)
FeCoNi_1.75_AlCu	2.1	54.4	(11)
FeCoNi_2_V_0.5_	1.62	70.3	(34)
FeCoNiMn	1.5	18.1	(35)
FeCoNiCrCu	33	29.6	(36)
FeCoNiCr	23	30.7	(36)
Fe_35_Co_20_Ni_20_Ti_20_Cr_5_	89	41.9	(37)
FeCoNiPd	0.04	116	(19)
FeCoNiPt	2	88	(19)
Fe_21_Co_25_Ni_32_Ti_22_ (400 K)	667	5.4	this work
Fe_21_Co_25_Ni_32_Ti_22_ (300 K)	489	10.8	this work
Fe_21_Co_25_Ni_32_Ti_22_ (200 K)	71	26.5	this work
Fe_21_Co_25_Ni_32_Ti_22_ (100 K)	5.3	40.0	this work
Fe_21_Co_25_Ni_32_Ti_22_ (50 K)	7.8	43.0	this work

We also comment on some Ni-based alloys that exhibit similar magnetic properties to FeCoNiTi. The multiphase Co–35Ni–20Cr–10Mo alloy MP35N shows FM ordering below 6.4 K in the as-cast state.^38^*H*_c_ reaches 100 Oe at 4.2 K, which increases to 200 Oe in the aged sample. The Cr addition might contribute to the enhanced *H*_c_, as seen in the cases of FeCoNiCrCu and FeCoNiCr. The low-temperature magnetization at a high field is comparable to *M*_s_ of FeCoNiTi. Antiferromagnetic coupling between CoNi and Cr is inferred. Compared to FeCoNiTi, *T*_c_ of MP35N is relatively low, which can be ascribed to the absence of Fe atoms. Haynes 242 alloy is a Ni–Mo–Cr superalloy widely used in the chemical industry.^39^ This alloy remains paramagnetic down to 3 K, likely due to the high content of Ni (∼70 at. %). This suggests that Ni content cannot be highly increased to achieve a high *T*_c_ value.

## Summary

We have investigated the magnetic properties of as-cast FeCoNiTi, an alloy manifesting dual-phase characteristics comprising the fcc and C14 Laves phases. Both phases exhibit FM ordering below *T*_c_ = 1084 K for the fcc phase and *T*_c_ = 214 K for the C14 phase. The C14 phase demonstrates the characteristics of a soft ferromagnet. Conversely, the fcc phase exhibits an *H*_c_ of 667 Oe at 400 K, classifying it as a high-coercive material within fcc HEAs based on FeCoNi. Notably, the *H*_c_ of the fcc phase experiences a pronounced reduction upon cooling below 300 K, concomitant with a rapid increase in *M*_s_ attributed to the onset of an additional FM state with *T*_c_ = 168 K. Electronic structure calculations for the fcc and C14 phases support the FM ground states in both phases. The *M*_s_ values at 50 K for both phases closely align with the theoretical magnetic moments. Finally, we delve into the underpinnings of the relatively high *H*_c_ observed at 300 or 400 K within the fcc phase. Our analysis underscores the limited influence of orbital moments on *H*_c_. Furthermore, an inferred negative correlation between *H*_c_ and *M*_s_ in fcc HEAs based on FeCoNi suggests a partial association with magnetocrystalline anisotropy. The discussion would be useful for manipulating the fundamental magnetic properties of fcc HEAs based on FeCoNi.
